# Unsupervised EEG Artifact Detection and Correction

**DOI:** 10.3389/fdgth.2020.608920

**Published:** 2021-01-22

**Authors:** Sari Saba-Sadiya, Eric Chantland, Tuka Alhanai, Taosheng Liu, Mohammad M. Ghassemi

**Affiliations:** ^1^Human Augmentation and Artificial Intelligence Lab, Department of Computer Science, Michigan State University, East Lansing, MI, United States; ^2^Neuroimaging of Perception and Attention Lab, Department of Psychology, Michigan State University, East Lansing, MI, United States; ^3^Computer Human Intelligence Lab, Department of Electrical & Computer Engineering, New York University Abu Dhabi, Abu Dhabi, United Arab Emirates

**Keywords:** electroencephalography, artifact rejection, brain computer interface, unsupervised learning, artifact removal

## Abstract

Electroencephalography (EEG) is used in the diagnosis, monitoring, and prognostication of many neurological ailments including seizure, coma, sleep disorders, brain injury, and behavioral abnormalities. One of the primary challenges of EEG data is its sensitivity to a breadth of non-stationary noises caused by physiological-, movement-, and equipment-related artifacts. Existing solutions to artifact *detection* are deficient because they require experts to manually explore and annotate data for artifact segments. Existing solutions to artifact *correction* or removal are deficient because they assume that the incidence and specific characteristics of artifacts are similar across both subjects and tasks (i.e., “one-size-fits-all”). In this paper, we describe a novel EEG noise-reduction method that uses representation learning to perform patient- and task-specific artifact detection and correction. More specifically, our method extracts 58 clinically relevant features and applies an ensemble of unsupervised outlier detection algorithms to identify EEG artifacts that are unique to a given task and subject. The artifact segments are then passed to a deep encoder-decoder network for unsupervised *artifact correction*. We compared the performance of classification models trained with and without our method and observed a 10% relative improvement in performance when using our approach. Our method provides a flexible end-to-end unsupervised framework that can be applied to novel EEG data without the need for expert supervision and can be used for a variety of clinical decision tasks, including coma prognostication and degenerative illness detection. By making our method, code, and data publicly available, our work provides a tool that is of both immediate practical utility and may also serve as an important foundation for future efforts in this domain.

## 1. Introduction

Electroencephalography (EEG) devices are pervasive tools used for clinical research, education, entertainment, and a variety of other domains (1). However, most EEG *applications* remain limited by the low signal to noise ratio inherent to data collected by EEG devices. EEG noise sources include movement artifacts, physiological artifacts (e.g., from perspiration), and instrument artifacts (resulting from the EEG device itself). While researchers have developed a number of methods to identify specific instances of these artifacts (2) in EEG data, most methods require manual labeling of exemplary artifact segments^1^ or special hardware, such as Electrooculography electrodes that are placed around the eyes, or large data-sets of templates, such as independent component scalp maps (3).

Manual annotation of artifacts in EEG data is problematic because it is time-consuming and may even be untenable if the specific profiles of artifacts in the EEG data vary as a function of the task, the subject, or the experimental trial within a given task for a given subject, as they so often do. These realities quickly scale the complexity of the artifact annotation problem and make the use of a one-size-fits-all artifact detection method infeasible for many practical use cases.

Even if artifacts could be identified with perfect fidelity, their simple removal (e.g., by deletion of the corrupted segment) may introduce secondary analytic complications that confound the performance of downstream methods that leverage these data. For instance, methods that rely on the stationarity of EEG segments will be confounded by simple removal of the artifact segments. Even the simplest approaches, such as averaging many EEG trials before extracting features (4), may be less effective if artifact occurrence is correlated with the trail type or experimental condition, thereby increasing the likelihood of a type II error and the consequent reduction in experimental power.

An essential challenge of artifact detection in EEG processing is that the definition of “artifact” depends on the specific task at hand. That is, a given EEG segment is an artifact if and only if it impacts the performance of downstream methods by manifesting as uncorrelated noise in a feature space that is relevant to those methods. For instance, muscle movement signatures confound comma-prognostic classification but are useful features for sleep stage identification (5).

The task-specific nature of artifacts makes their detection especially suitable for data-driven unsupervised approaches as the only requirement for the identification of artifacts using such methods is that the artifacts are *relatively* infrequent. That is, when mapping our data into feature spaces that are relevant to the specific EEG task, artifacts should stand out as rare anomalies. Indeed, many state-of-the-art approaches use unsupervised methods for the detection of specific artifact types under specific circumstances. For instance, the *Blink* algorithm described by Agarwal et al. is a fully unsupervised EEG artifact detection algorithm (6) that is effective for the detection of eye-blinks. While existing methods provide excellent performance for specific artifact types, there is a need for additional progress toward generalized artifact detection approaches, that make no assumptions about the task, subject, or circumstances.

It is also possible to go beyond artifact detection to *correct* the EEG trial by removing the artifact signal. EEG artifact removal is one instance of a more general class of noise reduction problems. The removal of noise from signal data has been a topic of scientific inquiry since Shannon laid the foundation for information theory in the 1940s (7); over the years, multiple signal processing approaches to this problem have found their way into EEG research. One such technique for artifact removal that is ubiquitous for EEG processing is Independent Component Analysis (ICA). This method and its modern derivative remain popular among the research community for unsupervised artifact correction. However, ICA still requires EEG experts to review the decomposed signals and manually classify them as either signal or noise. Furthermore, while ICA is undeniably an invaluable tool for many EEG applications, it also has limitations that are particularly poignant when the number of channels is low; ICA can only extract as many independent components as there are channels and will therefore be unable to isolate all independent noise components if the total number of independent noise components and signal sources exceeds the number of EEG electrodes (8).

Artifact removal is an especially common practice for a particular artifact type: the electrode “*pop*.” These artifacts result from abrupt changes in impedance, often due to loose electrode placement or bad conductivity (9, 10). Unlike muscle and movement artifacts, electrode pop is extremely localized, often affecting only one electrode channel. Channel interpolation is the process of replacing the signal of a corrupted channel with one that is interpolated from surrounding clean channels. Patrichella et al. demonstrated that knowing specific electrode locations (namely the exact electrode locations for each subject), and the distances between them can improve interpolation results (11, 12). However, this type of additional information is rarely available and often requires special dedicated hardware. Recently, Sadiya et al. proposed a deep learning convolutional auto-encoder based approach to learn task and subject-specific interpolation (13). By iteratively occluding channels in the input and using original data as the ground truth, the model learned how to interpolate channels in a self-supervised manner with no human annotation. Moreover, not only was the model able learn idiosyncratic information, such as subject-specific electrode location, beating state-of-the-art models, it was also possible to use transfer learning to improve performance on previously unseen tasks and subjects.

In this paper, we extend the aforementioned state-of-the-art approaches in artifact detection and rejection by building an end-to-end pipeline that solves both the detection and rejection problems together without making any assumptions concerning the task or artifact type.

Our artifact detection approach uses a collection of quantitative EEG features that are relevant for a wide variety of tasks including coma prognostics (14), diagnosing mental-illness (15), decoding mental representations (16), decoding attention deployment (17), and brain–computer interface design (18). Unsupervised outlier detection algorithms utilize these extracted features to identify artifacts in the EEG data. These unsupervised algorithms only require an estimate of the *frequency* of artifacts in the data, and can detect any artifact type, irrespective of the task. To guarantee that our results accurately represent the capabilities of these unsupervised outlier detectors we carefully selected algorithms that are qualitatively different from each other (for instance relying on local vs global characteristics of the data distributions) and explored hundreds of different possible configurations. Sub-section 2.2.1 provides a comprehensive review of the feature extraction process. Sub-section 2.2.2 details our experimentation with different outlier detection algorithms.

Our artifact correction approach uses a deep encoder-decoder network to correct artifacts that are *not restricted to only one channel*. Specifically, we frame our learning objective as a modified “*frame-interpolation*” task. Frame interpolation is the filling in of missing frames in a video (19). To the best of our knowledge, this is the first work that takes this approach to EEG artifact correction. The proposed approach is also unique in that it does not require the maintenance of any large data-set of templates or annotated data similarly to other state-of-the-art artifact removal methods (6). The model architecture as well as the exact objective formulation are discussed in detail in subsection 2.3.

The data-sets used in this work are discussed in detail in subsection 2.1. The results of the different experiments we conducted can be found in section 3. Finally, we discuss our findings, their broad implications, and the limitations of our approach in section 4.

## 2. Methods

In this paper, we propose an end-to-end pre-processing pipeline for the automated identification, rejection, and removal/correction of EEG artifacts using a combination of feature-based and deep-learning models which is intended for use as a general-purpose EEG pre-processing tool. To begin, we provide a brief overview of the data and methodological pipeline, calling out the specific subsections where the full details of each component of the pipeline are discussed.

**Figure 1:**
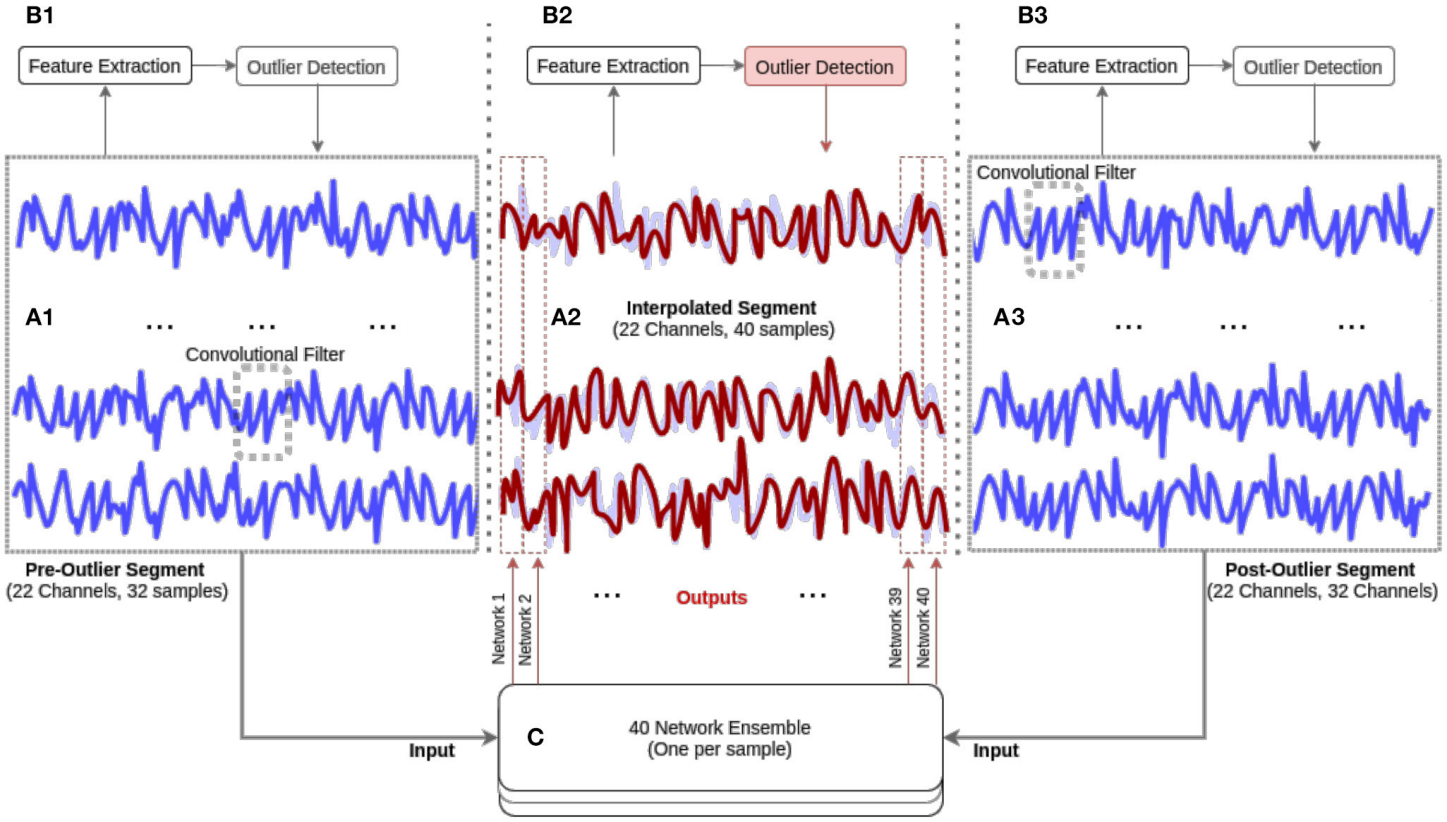
Our methodological approach. The EEG data is first segmented into epochs (see ***A*_1_**, ***A*_2_**, ***A*_3_**). Next, 58 features are extracted and an ensemble of unsupervised outlier detection methods are used (see ***B*_1_**, ***B*_2_**, ***B*_3_**) to identify EEG epochs that are artifact-ridden and require interpolation (see ***A***_**2**_ and ***B*_2_**). The artifact-ridden epochs are then interpolated by an ensemble of deep encoder-decoder networks (see red line in **C**).

In Figure 1 we provide a visualization of our proposed pre-processing pipeline; our method begins by performing unsupervised detection of epoched EEG segments in a 58-dimensional feature space (subsection 2.2). The trials that were not rejected in this initial stage are used to train a deep encoder-decoder network designed to correct artifacts segments (subsection 2.3).

While we demonstrate this method on a particular data set (described below), it is applicable (with no modifications) for any EEG pre-processing work. The methods are presented in the order of their processing within our proposed pipeline.

### 2.1. Data-Sets

#### 2.1.1. Data Acquisition

Our aim is to demonstrate that unsupervised anomaly detection is successfully used to identify artifacts in EEG data and that these artifacts can be corrected via representation learning methods (see section 2.3). To demonstrate the feasibility of our approach, it is necessary to not only have ground truth artifact annotations but also the ground truth labels for all trials, including those that were annotated as artifacts. While the artifact annotations allow us to test the unsupervised outlier detection methods, the trial labels allow us to verify that corrected EEG data can indeed be used in conjunction with that regular data for downstream analytic tasks (e.g., training a classification model). Unfortunately, available data sets usually do not contain rejected trials, and even when these annotations are available the original trial label is not included^2^. Therefore, our work is validated on two data-sets, hereinafter referred to as the *orientation* and *color* data-sets, that were previously collected by Saidya et al. (20). We briefly describe these datasets here; additional information about the data-sets is provided in the Supplementary Material.

Both experiments were passive viewing tasks. The orientation task stimulus consisted of 6 oriented gratings, the color task stimulus consisted of random dot fields in six different colors. The stimulus was generated using MGL, a library running in Matlab (Mathworks). The data was collected using a 32-electrode actiCHamp cap at 1,000 Hz. For each task, we collected data from seven subjects (four male) for a total of ~10,000 EEG Trials. All subjects reported normal or corrected to normal vision. The data were examined for noisy trials by expert annotators. Fully annotated and anonymized data-sets will be made available online. Participants gave informed consent and compensated at the rate of 15*$* per hour. The experimental procedures were approved by the Michigan State University Institutional Review Board and adhered to the tenets of the Declaration of Helsinki.

### 2.2. Unsupervised Artifact Detection

To benchmark the different outlier detection methods we collected a list of common features used in EEG research in different domains and applied various unsupervised outlier detection algorithms. Our main objective was to thoroughly investigate the feasibility of unsupervised artifact rejection for EEG.

#### 2.2.1. Feature Extraction

Building on the previous work of Ghassemi et al. (21), we reviewed the EEG literature and constructed a permissive list of several features that are commonly used for EEG classification tasks. In total, we identified and extracted 58 features. The code that extracts these features was written to allow for parallelization of the calculations and is accessible as a downloadable python 3.5 package^3^. See Table 1 for breakdown and references for all 58 features.

**Table 1:** EEG Features.

**Signal Descriptor**	**References**	**Brief description**
**Complexity features**		Degree of randomness or irregularity
Shannon entropy	(22)	Additive measure of signal stochasticity
Tsalis entropy (*n* = 10)	(23)	Non-additive measure of signal stochasticity
Information quantity (δ, α, θ, β, γ)	(24)	Entropy of a wavelet decomposed signal
Cepstrum coefficients (*n* = 2)	(25)	Rate of change in signal spectral band power
Lyapunov exponent	(26)	Separation between signals with similar trajectories
Fractal embedding dimension	(27)	How signal properties change with scale
Hjorth mobility	(28)	Mean signal frequency
Hjorth complexity	(28)	Rate of change in mean signal frequency
False nearest neighbor	(29)	Signal continuity and smoothness
ARMA coefficients (*n* = 2)	(30)	Autoregressive coefficient of signal at (t-1) and (t-2)
**Continuity features**		Clinically grounded signal characteristics
Median frequency		The median spectral power
δ band power		Spectral power in the 0–3 Hz range
θ band power		Spectral power in the 4–7 Hz range
α band power		Spectral power in the 8–15 Hz range
β band power		Spectral power in the 16–31 Hz range
γ band power		Spectral power above 32 Hz
Standard deviation	(31)	Average difference between signal value and it's mean
α/δ ratio	(14)	Ratio of the power spectral density in α and δ bands
Regularity (burst-suppression)	(14)	Measure of signal stationarity/spectral consistency
Voltage < (5, 10, 20 μ)		Low signal amplitude
Diffuse slowing	(32)	Indicator of peak power spectral density <8 Hz
Spikes	(32)	Signal amplitude exceeds μ by 3σ for 70 ms or less
Delta burst after spike	(32)	Increased δ after spike, relative to δ before spike
Sharp spike	(32)	Spikes lasting <70 ms
Number of bursts		Number of amplitude bursts
Burst length μ and σ		Statistical properties of bursts
Burst band powers (δ, α, θ, β, γ)		Spectral power of bursts
Number of suppressions		Segments with contiguous amplitude suppression
Suppression length μ and σ		Statistical properties of suppressions
**Connectivity features**		Interactions between EEG electrode pairs
Coherence – δ	(14)	Correlation in 0–4 Hz power between signals
Mutual information	(18)	Measure of dependence
Granger causality – All	(33)	measure of causality
Phase lag index	(34)	Association between the instantaneous phase of signals
Cross-correlation magnitude	(35)	Maximum correlation between two signals
Cross-correlation – lag	(35)	Time-delay that maximizes correlation between signals

*The 58 EEG features fell into three EEG signal property domains: Complexity features (25 in total), Category features (27 in total), Connectivity features (six in total)*.

These features can be grouped into three categories that measure the complexity, continuity, and connectivity of EEG activity. Before continuing to discuss our pipeline we will provide high-level intuition behind the inclusion of each category. We encourage the interested reader to refer to the previous work of Ghassemi et al. for a more detailed discussion of the specific features (21).

##### 2.2.1.1. Complexity Features (n = 25)

These features measure the complexity of the EEG signal from an information-theoretic perspective and are known to correlate with impaired cognitive functions and the presence of degenerative illnesses. Our first set of features is therefore a collection of information-theoretic complexity measures. Of special interest are the first three features shown in Table 1 as they are particularly prominent in EEG research: *Shannon's entropy* has been associated with neurological outcomes in post-anoxic coma patients (14); the entropy of the decomposed EEG wavelet signals (known as the *Subband Information Quantity*) have similarly been used in cardiac arrest studies (36, 37). *Tsalis entropy* is a generalization of Shannon's entropy that does not make assumptions about the independence of data channels (as Shannon's entropy does) and has been shown to be particularly useful for the characterization of complexity in EEG data (23).

##### 2.2.1.2. Continuity features (n = 27)

These features capture the regularity and volatility of EEG activity. Bursts, spikes, and unusual changes in the mean and standard deviation in the frequency and power domains are examples of continuity features that are relevant for a variety of clinical tasks. See Hirsh et al. for an in-depth review of continuity and it's relevance to clinical care (38).

##### 2.2.1.3. Connectivity features (n = 6)

. These features reflect the statistical dependence of EEG signal activity *across* two or more channels. Functional connectivity networks are established features of normal brain functioning. We draw on the rich literature on measuring connectivity from EEG signals (39) extracting features that have previously been used for designing brain computer interfaces (18) as well as in mental illness, perception, and attention research [see (15), (16), and (17), respectively].

#### 2.2.2. Outlier Detection Methods

We explored a set of ten algorithms for unsupervised artifact detection; the explored algorithms were inspired by the work of Zhao et al. (40). The algorithms can be divided into two general groups: statistical methods and representation learning methods; they are described in more detail in the “*Statistical Methods*” and “*Representation Learning Based Methods*” sections below. The hyper-parameters of each method were determined by randomly exploring the hyper-parameter space and choosing the settings that yielded the best performance of the methods on the data according to our artifact annotations.

##### 2.2.2.1. Statistical methods

Statistical methods identify anomalies based on statistical measures extracted from the data, thereby producing an “anomaly score” for each trial. The Histogram-Based Outlier detection (HBOS) method uses histograms with dynamic bin widths to detect clusters and anomalies in different feature dimensions. Despite the simplicity of the approach it has been shown to work well on a variety of data types (41). The Local Outlier Factor (LOF) method similarly calculates an “outlier score”; however, instead of global measures, it relies on the local density of the data as it's main indicator (42). Another popular local algorithm, the Angle-Based Outlier Detector (ABOD), calculates the cosine similarity of data points with their neighbors and uses the variance of these scores to generate anomaly scores (43). Finally, we also trained a One Class SVM Detector (OCSVM), a classic algorithm for outlier detection (44). In this algorithm, an SVM is trained on the entire data-set and afterwards every instance is scored based on its distance from the class boundary; the intuition is that the infrequent outliers will contribute less to the decision boundary calculation and will be more likely to be on the margin of the learned boundary.

As previously mentioned, we selected these detectors to be different in the type of statistical measurements they use. Therefore, it makes sense to also train ensemble classifiers to further improve the outlier detection accuracy. Specifically, we trained five hundred *Locally Selective Combination in Parallel* (LSCP) Outlier Ensembles (45) with different combinations of the algorithms mentioned above.

##### 2.2.2.2. Representation learning based methods

Unlike statistical methods, representation-learning-based outlier detectors do not simply calculate statistical properties of featurized data. The most basic classifier uses auto-encoder (AUTO) based deep learning architectures to learn a lower-dimensional representation of the data that enables the best possible reconstruction of the original signal; the embedding would be optimized for the common regular data points thereby producing distinctly noisy reconstructions for the outlier trials (46). This classifier can be viewed as a modern update of similar classic outlier detection methods that use methods, such as PCA reconstruction instead of training a deep auto-encoder (PCA) (47). A more sophisticated approach uses Variational Auto-Encoders (VAE). This class of algorithms tries to ensure that the learned embedding captures the structure of the original data by penalizing the classifier if the embedding does not follow a standard normal distribution (48). Finally, we also examine a Generative Adversarial Active Learning (GAAL) outlier detector (49), which uses generative adversarial networks to generate outliers. This method can be used to improve any of the statistical methods described in 2.2.2.1. We also use an extension of the original method to learn multiple generators (MGAAL).

### 2.3. Artifact Correction

As previously mentioned, encoder-decoder based deep learning methods have proven useful for channel interpolation (13). In this section we discuss an extension of this approach that utilizes the same framework for artifact correction. Namely, given an EEG data segment with an isolated artifact we remove the corrupted segment and use the data samples preceding and proceeding it to fill in the resulting void. This problem is equivalent to the “*frame-interpolation*” task of filling in missing frames in a video (19).

#### 2.3.1. The Model

##### 2.3.1.1. Input representation

The channel interpolation model proposed in Saba-Sadiya et al. (13) represented the EEG as a time series of 2D topologically organized arrays. This reflects the spatial nature of the EEG channel interpolation issue; the interpolated values at different time points are treated as independent. To the best of the author's knowledge, this is a standard assumption for EEG interpolation algorithms. For instance, Petrichella et al. and Courellis et al. calculate the interpolated values of the missing data at each time point separately (11, 12). However, research on convolutional neural networks for EEG decoding and visualization have shown performance benefits from presenting the input as a column of electrodes unfolding in time, as this facilitates the learning of temporal modulations (50). Since artifact correction is first and foremost a process of completing gaps across time we decided to depart from Saba-Sadiya et al. (13) and use a 2D array representation with the number of time steps as the width of the array.

##### 2.3.1.2. Architecture

The best frame interpolation models involve calculating object trajectory and accounting for possible occlusion (e.g., if one object moves behind another). With these “flow computations” and a stack of the frames before and after the missing image a convolutional encoder-decoder can generate realistic intermediate images (19). Unlike video, EEG data have only one spatial dimension (see subsection 2.3.1.1) and are not analogous to local phenomena, such as occlusion or object movement; these can occur as EEG modulations and are often thought of as mostly global in nature (50). Therefore, we only concern ourselves with a stacked convolutional auto-encoder. This architecture is shared by previously discussed state-of-the-art algorithms for both frame interpolation and channel interpolation (13, 50).

The interpolation of each frame is done separately, thus to predict *n* frames it is necessary to train *n* networks. Technically this is equivalent to training one ensemble model, however, by separating the networks we allow for easier parallelization of the training process. Specifically, given a series of EEG frames *x*_1_, *x*_2_, …, *x*_*n*_ where *x*_*t*_ is a vector of all the channel values at time *t*, and assuming that the series is missing all frames between time points *t*_*b*_ and *t*_*e*_, our network learns to predict *x*_*t*_*q*__ from the two stacks, *x*_*t*_*b*_−*h*_, *x*_*t*_*b*_−*h*+1_, …, *x*_*t*_*b*__ and *x*_*t*_*e*__, *x*_*t*_*e*_+1_, …, *x*_*t*_*e*_+*h*_ where *t*_*q*_ ∈ (*t*_*b*_, *t*_*e*_) and *h* is some small positive integer representing how many frames before and after the missing segment can be perceived. Every network is trained to predict the value at one specific value of *q*. Every network takes the same 2*h* frames (half preceding the missing segment and half following it) to calculate the value at a given frame.

### 2.4. Model Validation Approach

#### 2.4.1. Artifact detection method

The performance of the artifact detection methods was assessed by inspecting the agreement between the artifact detection approach and the expert annotations from the two data sets (color and orientation). More specifically, the agreement was measured using the f-score and Cohen's Kappa (first and second values in each cell, respectively). We compared the performance of our model against the expected performance of a classifier with knowledge of the exact number of artifacts; this random classifier is expected to have an f-score of 0.172 and a Kappa of 0.029. We ran the detection algorithms in two configurations, for each subject separately and for the entire aggregated data. We hypothesize that the performance will drop when using the aggregated configuration, as each individual setup for an EEG recording is likely to introduce unique artifacts (due to loose connections or subject-specific circumstances, such as perspiration).

#### 2.4.2. Artifact correction method

To optimize the parameters of the artifact correction model, we produced training data from trials that were marked as artifacts free by our unsupervised artifact detection method (section 2.2.2) and randomly removed a segment from the middle of the trial. The *h* samples proceeding the removed segment and *h* samples preceding it were used as input for the model while the removed segment was the ground truth (*h* was a hyper-parameter optimized on the training set). For the purposes of validating the artifact correction model, all EEG data were re-sampled to 200*Hz*. The reconstructed segments were 200*ms* each.

#### 2.4.3. End-to-end assessment approach

We ran a number of tests to examine if the trials reconstructed by our artifact correction method could be used to enhance the performance of downstream EEG tasks. More specifically, we trained two SVM models to predict the label of the trial from the color data-set: one SVM was trained using the *raw data*, and the other was trained using the raw data *after artifact correction*. Both models were validated using 5-fold cross-validation, and the performance of the models on the test set (μ and σ) was reported.

We also evaluated the impact of our artifact correction method on downstream EEG tasks when applied to *clean trials, exclusively*; this evaluation allowed us to test for inadvertent degeneration in signal quality of clean segments when processed by our method. More specifically, we applied our artifact correction method to 20% of *clean* trials and used the resulting data to train an additional SVM model.

## 3. Results

This section presents the results of the two main components in our pipeline, the artifact detection method and the artifact correction method on the data described in 2.1.

### 3.1. Artifact Detection Results

In Table 2, we compare the *average* performance of the outlier detection methods described in section 2.2.2 when applied to each subject *separately*. Therefore, each value is the mean of the algorithm's performance across subjects. As previously mentioned, the expected performance of a baseline random classifier with knowledge of the exact number of artifacts is an f-score of 0.172 and a Kappa of 0.029. Hence, all models other than the *ABOD* classifier performed significantly better than the baseline (one tailed *t*-test with a *p* = 0.05 significance level).

**Table 2:** Comparison of the different unsupervised outlier detection methods when applied to each subject separately.

**Statistical methods**	**HBOS**	**LOF**	**ABOD**	**OCSVN**	**LSCP**
Orientation	0.564 0.473	0.218 0.065	0.11 0.06	0.41 0.29	0.577 0.489
Color	0.5 0.4	0.241 0.091	0.1 −0.08	0.36 0.23	0.51 0.411
**Representation learning**	**AUTO**	**PCA**	**VAE**	**GAAL**	**MGAAL**
Orientation	0.53 0.44	0.527 0.426	0.477 0.368	0.429 0.311	0.428 0.309
Color	0.51 0.42	0.477 0.367	0.478 0.368	0.241 0.086	0.389 0.263

*We calculated the mean f-score and Cohen's Kappa (first and second row in every cell) across all subject. HBOS, Histogram based outlier detection; LOF, Local outlier factor Method; ABOD, Angle-based outlier detector; OCSVM, One class support vector machine; LSCP, Locally selective combination of parallel outlier Ensembles; AUTO, Auto-encode based method; VAE, Variational auto-encoder based method; GAAL, Generative Adversarial Active Learning; MGAAL, Multi-object Generative Adversarial Active Learning*.

Unsurprisingly, the best outlier detector was an *LSCP* ensemble classifier that performed 16.86*x* better than the baseline method, and 1.03*x* better than the next best approach; the best performing configuration of the classifier consisted of two *HBOS* classifiers and one *OCSVM*. While it is difficult to interpret ensemble classifiers it is worth noting that the two histogram-based classifiers diverged quite substantially; one using a high number of histogram bins and a rigid outlier scoring policy (*tol* = 0.1) while the other using a smaller number of bins and more relaxed policy (*tol* = 0.5). A simple auto-encoder was the best representation learning algorithm, closely followed by the PCA algorithm. We speculate that the auto-encoder could have possibly had better performance if more data were available for each subject. See our Supplementary Material for a breakdown of trial and artifact numbers for each subject.

In Table 3, we compare the performance of the outlier detection methods described in section 2.2.2 when applied to the subjects *aggregated* data; that is, subject were not considered separately as they were in the results from Table 2. When compared to the results shown in Table 2, the performance decreased for most models. This is not surprising as the fundamental assumption of unsupervised methods is that the data are homogeneous with the exceptions of the outliers. Here again, the LSCP method performed the best of the tested approaches. A comparison of the results in Tables 2, 3 provide motivation for the development of subject-specific anomaly detection approaches. Moreover, the comparison also highlights that the unsupervised algorithms and the features we extracted can successfully capture both common EEG artifacts and subject-specific idiosyncrasies.

**Table 3:** The performance of the models trained on data aggregated from all the subjects.

**Statistical methods**	**HBOS**	**LOF**	**ABOD**	**OCSVN**	**LSCP**
Orientation	0.502 0.4	0.246 0.095	0.07 −0.11	0.362 0.234	0.537 0.441
Color	0.476 0.35	0.305 0.15	0.09 −0.108	0.377 0.238	0.463 0.332
**Representation learning**	**AUTO**	**PCA**	**VAE**	**GAAL**	**MGAAL**
Orientation	0.488 0.338	0.448 0.338	0.447 0.336	0.383 0.246	0.393 0.258
Color	0.414 0.283	0.437 0.312	0.436 0.31	0.185 0.022	0.393 0.258

*The f-score and Cohen's Kappa are presented in the first and second row in every cell*.

### 3.2. Artifact Correction Results

#### 3.2.1. Network Optimization

Our first step was to optimize the network hyper-parameter configurations. This included testing different sizes of both the layers and convolution filter, as well as exploring different hyper-parameters, such as optimization algorithms, dropout rates, and activation functions. To train the network we followed the method discussed in section 2.2.2: we randomly extracted 104 samples from the data, the first and last 32 samples were stacked and used as the input to the model, and the sample at position *i* from the remaining 40 samples was used as the ground truth. Essentially we are training a network to predict the values after removing 40 samples (200*ms*) using the 32 samples that before and after the removed segment. The best performing network (lowest loss) was different for different *t*s. The optimal topology for reconstructing sample 20 is available in the Supplementary Material as a reference of the type of convolutional U-net architecture used.

#### 3.2.2. End-to-End Assessment

In Table 4 we compare the classification accuracy of a 5-fold SVM model trained to perform a downstream classification of trial type using down-sampled EEG data with three different configurations of the data: (1) the raw EEG data, (2) the data after correction of artifact segments, and (3) the data following “correction” of a random 40 samples of 20% of the non-artifact segments. Note that while simple this type of analysis is used in actual EEG research (4).

**Table 4:** Mean accuracies of simple SVM classifiers.

	**Original EEG**	**EEG with random correction**	**EEG with artifact correction**
All trials	0.3	0.31	0.33
Rejected trials	0.23	0.23	0.29

*A simple t-test confirmed that all accuracies were significantly above chance level (1/6 for six different colors) at a p = 0.05 level. Original EEG: The Original EEG data. EEG with Random Correction: The EEG data after random artifact free trials were “corrected.” EEG with artifact correction: The data after we applied the EEG artifact correction on the trials that were marked as artifact ridden*.

The performance remained comparable after using the artifact correction on trials that did not contain any artifacts. This is a strong indication that the model is indeed able to learn how to reconstruct the original EEG signal. When using the corrected trials with EEG artifacts the classification accuracy improved by 10% overall and over 20% for trials that were marked as containing artifacts. These results successfully demonstrate that our unsupervised end-to-end artifact correction pipeline improves down-stream analysis.

## 4. Discussion

### 4.1. Significance of Our Results

In this paper, we presented an end-to-end pipeline that is capable of unsupervised artifact detection and correction. Our results demonstrate that data-driven approaches for unsupervised outlier detection can be extremely useful when applied to the problem of EEG artifact detection. Interestingly, the classifiers with the best performance (HBOS, OCSVM, and the best performing LSCP) are global classifiers; this might indicate that EEG artifacts are better discriminated by global characteristics. This supports our previous observation that artifacts are task specific and infrequent occurrences of uncorrelated noise. It is worth noting that, as demonstrated in Table 3, the classifiers we trained were able to learn subject-specific idiosyncrasies.

While the accuracy and agreement between the annotators and the detectors were far from perfect, the Cohen Kappa of the best performing algorithm was comparable to the inter-rater agreement levels of expert annotators reported in the literature; for instance, when asked to annotate, “periodic discharges” (a specific type of artifact) and “electrographic seizure” annotators had a Cohen's Kappa of 0.38 and 0.58, respectively (51). Our results indicate that an unsupervised outlier detection is a feasible approach for generalized EEG artifact detection.

### 4.2. The Data-Sets

We validated our framework on two novel data-sets. To test the impact of artifact correction algorithms on downstream analysis it is necessary to have ground truth artifact annotation as well as knowledge of the labels of all trials, including those that are artifact ridden. Unfortunately, public data-sets often exclude trials that contain artifacts. Even in the rare occasions in which these trials are made available, the labels are often replaced with a special identifier for rejected trials^4^. We hope our data-sets inspire other researchers to adopt more thorough data publishing practices as data-availability is perhaps the primary limiting factor in artifact correction research.

### 4.3. The Strength of Unsupervised End-to-End Methods

The accuracy of simple classifiers improved modestly after artifact removal. It is possible that replacing our deep-learning-based artifact removal components with an ICA artifact removal algorithm (52) could yield better results. However, two important distinctions should be made: First, the proposed method does sidestep many weaknesses inherent to ICA (8) (such as the number of independent components being limiting by the number of channels, which is particularly problematic for lightweight commercial EEG setups). Secondly, while the independent component deconstruction itself is data driven and unsupervised, the ICA method still requires visual inspection and analysis of the decomposed signal by human experts. In contrast, our method can be put into effect without any human intervention, making it is suitable for online EEG applications or as a no-cost first step before a more thorough analysis. In general, supervised methods unquestionably out-perform unsupervised ones and we fully acknowledge that the pipeline proposed in this work is no different. It is therefore useful to consider unsupervised methods not as replacements of currently existing algorithms but as complimentary additions to the toolbox of the EEG researcher. With this in mind, we intentionally designed our end-to-end pipelines to be highly modular; An experienced researcher can easily substitute our last component with an ICA artifact removal algorithm, and in contrast, researchers that have access to artifact annotations (for instance by virtue of employing specialized hardware during data acquisition) will be able to use their method in conjunction with ours or sidestep the first processes completely and apply only the artifact correction component before carrying on with the analysis process.

### 4.4. Limitations

We did not formally evaluate the reconstruction performance of the model because (1) there is not an authoritative literature baseline, and (2), insofar as the reconstruction enhances the ability of the downstream classification model to perform their intended classification tasks, the reconstruction is valid and valuable. There are a few limitations that we hope to address in future work. First and foremost, this artifact detection method can only be used if the frequency of the artifacts is low enough for them to be considered outliers. While this is indeed the case for the vast majority of EEG use cases, tasks, such as seizure detection often involve long periods of unusually low signal to noise ratio. Additionally, the performance of our artifact correction network would likely benefit from introducing more complex component into the architecture. For instance, introducing temporal dependencies via an LSTM component would guarantee that the corrected frame at time *t* influences the frame at time *t*+1. Finally, our method is in dire need of being validated on additional tasks and data-sets.

Despite the challenges described above, we believe that our work demonstrates the feasibility of an EEG pre-processing pipeline which if adopted could facilitate and expedite the often tenuous process of artifact annotation and removal, and could therefore be extremely beneficial for the general EEG research community.

## 5. Conclusion and Future Work

The applications of EEG are numerous and diverse, and while this impacts the particularities of what components are classified as part of the signal vs. artifacts, data homogeneity is a common concern in this area of research. Building on this data science perspective, in this work we appropriated state-of-the-art data-driven methods to construct an end-to-end unsupervised pipeline for general artifact detection and correction. We introduced two new data-sets and demonstrated that the inter-rater reliability of our artifact detection component against expert annotators is comparable to reported inter-human levels. Furthermore, we demonstrated how applying the complete pipeline on a data-set can improve the performance of common downstream analysis. The pipeline makes use of a wide range of handcrafted clinically relevant features, and we believe the released python package will be of use to many in the EEG research community.

## Data Availability Statement

The raw data supporting the conclusions of this article will be made available by the authors, without undue reservation.

## Ethics Statement

The studies involving human participants were reviewed and approved by The Michigan State University Human Research Protections Program IRB. The participants provided their written informed consent to participate in this study.

## Author Contributions

SS-S: data collection and annotation, coding for the Methods section, and writing. EC: data collection and annotation, helped code for the Methods section, and article review. TA: algorithm design and writing. TL: coded the experiment and provided the EEG equipment used for data collection. MG: literature review for, and design of, the models presented in the Methods section, and writing. All authors contributed to the article and approved the submitted version.

## Conflict of Interest

The authors declare that the research was conducted in the absence of any commercial or financial relationships that could be construed as a potential conflict of interest.

## Footnotes

^1^Which may be used as “templates” by statistical or rule-based methods for the identification (and potential rejection) of noisy data epochs.

^2^For instance BCI competitions data: http://bbci.de/competition/.

^3^Code available at: https://github.com/sari-saba-sadiya/EEGExtract.

^4^For an example of standard EEG publishing practices see the BCI Competition data-sets.

## References

[1] Siripornpanich V Sampoon K Chaithirayanon S Kotchabhakdi N Chutabhakdikul N. Enhancing brain maturation through a mindfulness-based education in elementary school children: a quantitative EEG study. Mindfulness. (2018) 9:1877–84. 10.1007/s12671-018-0930-3

[2] Urigüen JA Garcia-Zapirain B. EEG artifact removal—state-of-the-art and guidelines. J Neural Eng. (2015) 12:031001. 10.1088/1741-2560/12/3/031001 25834104

[3] Shamlo NB Kreutz-Delgado K Kothe C Makeig S. EyeCatch: data-mining over half a million EEG independent components to construct a fully-automated eye-component detector. In: 2013 35th Annual International Conference of the IEEE Engineering in Medicine and Biology Society (EMBC) (Osaka) (2013). p. 5845–8. 10.1109/EMBC.2013.6610881 PMC4136453 24111068

[4] Cichy RM Ramirez FM Pantazis D. Can visual information encoded in cortical columns be decoded from magnetoencephalography data in humans? Neuroimage. (2015) 121:193–204. 10.1016/j.neuroimage.2015.07.011 26162550

[5] Ghassemi MM Moody BE Lehman LH Song C Li Q Sun H. You snooze, you win: the PhysioNet/Computing in Cardiology challenge 2018. In: 2018 Computing in Cardiology Conference (CinC), Vol. 45 (Maastricht), (2018). p. 1–4. 10.22489/CinC.2018.049 PMC8596964 34796237

[6] Agarwal M Sivakumar R. Blink: a fully automated unsupervised algorithm for eye-blink detection in EEG signals. In: 2019 57th Annual Allerton Conference on Communication, Control, and Computing (Allerton) (Monticello, MN), (2019). p. 1113–21. 10.1109/ALLERTON.2019.8919795

[7] Shannon CE. Communication in the presence of noise. Proc IRE. (1949) 37:10–21. 10.1109/JRPROC.1949.232969

[8] Djuwari D Kumar D Palaniswami M. Limitations of ICA for artefact removal. Conf Proc IEEE Eng Med Biol Soc. (2005) 5:4685–8. 10.1109/IEMBS.2005.1615516 17281286

[9] Walczak T Chokroverty S. Electroencephalography, Electromyography, and Electro-Oculography: General Principles and Basic Technology. Elsevier Inc. (2009).

[10] Britton JW Frey LC Hopp JL Korb P Koubeissi MZ Lievens WE. Electroencephalography (EEG): An Introductory Text and Atlas of Normal and Abnormal Findings in Adults, Children, and Infants [Internet]. St. Louis EK Frey LC editors. Chicago: American Epilepsy Society (2016). 27748095

[11] Petrichella S Vollere L Ferreri F Guerra A Määtta S Könönen M. Channel interpolation in TMS-EEG: a quantitative study towards an accurate topographical representation. In: 2016 38th Annual International Conference of the IEEE Engineering in Medicine and Biology Society (EMBC) (Florida) (2016). p. 989–92. 10.1109/EMBC.2016.7590868 28268490

[12] Courellis HS Iversen JR Poizner H Cauwenberghs G. EEG channel interpolation using ellipsoid geodesic length. In: 2016 IEEE Biomedical Circuits and Systems Conference (BioCAS) (Shanghai) (2016). p. 540–3. 10.1109/BioCAS.2016.7833851

[13] Saba-Sadiya S Alhanai T Liu T Ghassemi M. EEG channel interpolation using deep encoder-decoder networks. In: 2020 IEEE International Conference on Bioinformatics and Biomedicine (BIBM) (Seoul) (2020).

[14] Tjepkema-Cloostermans M van Meulen F Meinsma G van Putten M. A cerebral recovery index (CRI) for early prognosis in patients after cardiac arrest. Crit Care. (2013) 17:R252. 10.1186/cc13078 24148747 PMC4056571

[15] Uhlhaas P Singer W. Abnormal neural oscillations and synchrony in schizophrenia. Nat Rev Neurosci. (2010) 11:100–13. 10.1038/nrn2774 20087360

[16] Hipp J Engel A Siegel M. Oscillatory synchronization in large-scale cortical networks predicts perception. Neuron. (2011) 69:387–96. 10.1016/j.neuron.2010.12.027 21262474

[17] Zion Golumbic E Cogan GB Schroeder CE Poeppel D. Visual input enhances selective speech envelope tracking in auditory cortex at a “Cocktail Party”. J Neurosci. (2013) 33:1417–26. 10.1523/JNEUROSCI.3675-12.2013 23345218 PMC3711546

[18] Ang KK Chin ZY Zhang H Guan C. Mutual information-based selection of optimal spatial-temporal patterns for single-trial EEG-based BCIs. Pattern Recogn. (2012) 45:2137–44. 10.1016/j.patcog.2011.04.018

[19] Jiang H Sun D Jampani V Yang MH Learned-Miller E Kautz J. Super SloMo: high quality estimation of multiple intermediate frames for video interpolation. In: 2018 IEEE/CVF Conference on Computer Vision and Pattern Recognition (Salt Lake City, UT) (2018). 10.1109/CVPR.2018.00938

[20] Saba-Sadiya S Chantland E Liu T. Decoing EEG From Passive Viewing. GitHub (2020). Available online at: https://github.com/sari-saba-sadiya/DEPV

[21] Ghassemi MM. Life After Death: Techniques for the Prognostication of Coma Outcomes After Cardiac Arrest. Cambridge, MA: Massachusetts Institute of Technology (2018).

[22] Shannon CE Weaver W. The Mathematical Theory of Communication. Champaign, IL: University of Illinois Press (1998).

[23] Geocadin R Muthuswamy J Sherman D Thakor N Hanley D. Early electrophysiological and histologic changes after global cerebral ischemia in rats. Mov Disord. (2000) 15:14–21. 10.1002/mds.870150704 10755267

[24] Shin HC Jia X Nickl R Geocadin RG Thakor Ast NV. A subband-based information measure of EEG during brain injury and recovery after cardiac arrest. IEEE Trans Biomed Eng. (2008) 55:1985–90. 10.1109/TBME.2008.921093 18632361 PMC3050511

[25] Oppenheim AV Schafer RW. From frequency to quefrency: a history of the cepstrum. IEEE Signal Process Mag. (2004) 21:95–106. 10.1109/MSP.2004.1328092

[26] Wolf A Swift JB Swinney HL Vastano JA. Determining Lyapunov exponents from a time series. Phys D Nonlin Phen. (1985) 16:285–317. 10.1016/0167-2789(85)90011-9

[27] Accardo A Affinito M Carrozzi M Bouquet F. Use of the fractal dimension for the analysis of electroencephalographic time series. Biol Cybern. (1997) 77:339–50. 10.1007/s004220050394 9418215

[28] Oh SH Lee YR Kim HN. A novel EEG feature extraction method using Hjorth parameter. Int J Electron Electric Eng. (2014) 2:106–10. 10.12720/ijeee.2.2.106-110

[29] Hegger R Kantz H. Improved false nearest neighbor method to detect determinism in time series data. Phys Rev E. (1999) 60:4970. 10.1103/PhysRevE.60.4970 11970367

[30] Box GE Jenkins GM Reinsel GC Ljung GM. Time Series Analysis: Forecasting and Control. Hoboken, NJ: John Wiley & Sons (2015).

[31] Ross SM. Introductory Statistics. Cambridge, MA: Academic Press (2017).

[32] Stern JM. Atlas of EEG Patterns. Philadelphia, PA: Lippincott Williams & Wilkins (2005).

[33] Blinowska KJ Kuś R Kamiński M. Granger causality and information flow in multivariate processes. Phys Rev E. (2004) 70:050902. 10.1103/PhysRevE.70.050902 15600583

[34] Stam C Nolte G Daffertshofer A. Phase lag index: assessment of functional connectivity from multi channel EEG and MEG with diminished bias from common sources. Hum Brain Mapp. (2007) 28:1178–93. 10.1002/hbm.20346 17266107 PMC6871367

[35] Kay SM. Fundamentals of Statistical Signal Processing. Upper Saddle River, NJ: Prentice Hall PTR (1993).

[36] Shin H Tong S Yamashita S Jia X Geocadin G Thakor N. Quantitative EEG and effect of hypothermia on brain recovery after cardiac arrest. IEEE Trans Biomed Eng. (2006) 53:1016–23. 10.1109/TBME.2006.873394 16761828 PMC3050568

[37] Jia X Koenig M Nickl R Zhen G Thakor N Geocadin R. Early electrophysiologic markers predict functional outcome associated with temperature manipulation after cardiac arrest in rats. Crit Care Med. (2008) 36:1909. 10.1097/CCM.0b013e3181760eb5 18496359 PMC2849160

[38] Hirsch LJ LaRoche SM Gaspard N Gerard E Svoronos A Herman ST. American Clinical Neurophysiology Society's standardized critical care EEG terminology: 2012 version. J Clin Neurophysiol. (2013) 30:1–27. 10.1097/WNP.0b013e3182784729 23377439

[39] Schoffelen J Gross J. Source connectivity analysis with MEG and EEG. Hum Brain Mapp. (2009) 30:1857–1865. 10.1002/hbm.20745 19235884 PMC6870611

[40] Zhao Y Nasrullah Z Li Z. PyOD: a python toolbox for scalable outlier detection. J Mach Learn Res. (2019) 20:1–7.

[41] Goldstein M Dengel A. Histogram-Based Outlier Score (HBOS): A Fast Unsupervised Anomaly Detection Algorithm. Saarbrücken: German Research Center for Artificial Intelligence (2012) 59–63.

[42] Breunig MM Kriegel H Ng RT Sander J. LOF: identifying density-based local outliers. SIGMOD Rec. (2000) 29:93–104. 10.1145/335191.335388

[43] Kriegel H Schubert M Zimek A. Angle-based outlier detection in high-dimensional data. In: Proceedings of the 14th ACM SIGKDD International Conference on Knowledge Discovery and Data Mining (Las Vegas, NV) (2008). p. 444–52. 10.1145/1401890.1401946

[44] Schölkopf B Platt JC Shawe-Taylor J Smola AJ Williamson RC. Estimating the support of a high-dimensional distribution. Neural Comput. (2001) 13:1443–71. 10.1162/089976601750264965 11440593

[45] Zhao Y Hryniewicki MK Nasrullah Z Li Z. LSCP: locally selective combination in parallel outlier ensembles.1 In: SDM (Calgary, AB) (2019). 10.1137/1.9781611975673.66

[46] Aggarwal CC. Outlier analysis. In: Data Mining: The Textbook. Cham: Springer International Publishing (2015). p. 75–9. 10.1007/978-3-319-14142-8_8

[47] Shyu ML Chen SC Sarinnapakorn K Chang LW. A Novel Anomaly Detection Scheme Based on Principal Component Classifier. AD-a465 712. Coral Gables, FL: University of Miami, Department of Electrical and Computer Engineering (2003). Available online at: https://books.google.com/books?id=iXEinQAACAAJ

[48] An J Cho S. Variational Autoencoder Based Anomaly Detection Using Reconstruction Probability. (2015).

[49] Liu Y Li Z Zhou C Jiang Y Sun J Wang M. Generative adversarial active learning for unsupervised outlier detection. In: Proceedings of the IEEE Transactions on Knowledge and Data Engineering. (2019). 10.1109/TKDE.2019.2905606

[50] Schirrmeister RT Springenberg JT Fiederer LDJ Glasstetter M Eggensperger K Tangermann M. Deep learning with convolutional neural networks for EEG decoding and visualization. Hum Brain Mapp. (2017) 38:5391–420. 10.1002/hbm.23730 28782865 PMC5655781

[51] Halford JJ Shiau D Desrochers JA Kolls BJ Dean BC Waters CG. Inter-rater agreement on identification of electrographic seizures and periodic discharges in ICU EEG recordings. Clin Neurophysiol. (2015) 126:1661–9. 10.1016/j.clinph.2014.11.008 25481336 PMC4439396

[52] Jung TP Makeig S Humphries C Lee TW McKeown M Iragui V. Removing electroencephalographic artifacts by blind source separation. Psychophysiology. (2000) 37:163–78. 10.1111/1469-8986.3720163 10731767

