# Supplementary material for: Unsupervised EEG Artifact Detection and Correction
Source: Front Digit Health. 2021 Jan 22;2:608920. doi: 10.3389/fdgth.2020.608920 (PMC8521924; doi:10.3389/fdgth.2020.608920)
Supplement: Supplementary file 1 [file Data_Sheet_1.PDF]

Supplementary Material

1 DATA COLLECTION

The two data-sets used in this study were originally collected to test different EEG decoding algorithms. In this section of the supplementary materials provides a detailed overview of the task and the data acquisition process.

1.1 Stimuli

The two experiments were both passive viewing tasks, each involving six different stimuli. The subjects were directed to fixate at a fixation cross in the middle of the screen. Each trial consisted of $1000ms$ of stimulus followed by $1000ms - 1500ms$ of inter trial interval. The data from the inter trial interval was discarded. The orientation stimulus consisted of 6 oriented gratings with equal angle differences. The color stimulus consisted of random dot fields in 6 different colors. To eliminate potential confounds, each subject adjusted the screen to achieve isoluminance between all colors. In total there were 648 trials per experiment per subject (108 trials per condition). We collected additional trials for some subject but kept the balance between the different trial types. See Table S1 for a subject by subject breakdown of the number of trials and artifacts.

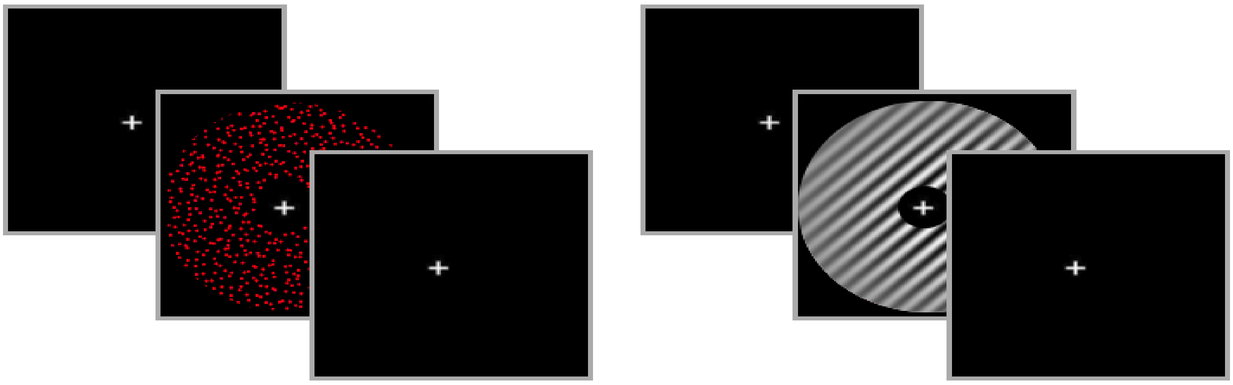

Figure S1. The fixation cross appears for 200ms before a colored random dot field (or a gabor patch) appears around the fixation. A *blink* trial is introduced between every two to four regular trials to allow the participant to relax their eyes.

Table S1. Breakdown of the number of trials and artifact in the two data-sets of each subject

Subject Number	Color Data		Orientation Data	
	Trials	Artifacts	Trials	Artifacts
1	648	82	648	97
2	669	139	681	132
3	648	106	648	87
4	669	55	648	53
5	648	84	648	81
6	648	30	648	31
7	648	132	648	89

1.2 Data Acquisition

Continuous EEG activity was recorded using actiCHamp system with BrainVision recorder software. The participants were fitted with a 64 channel actiCap with active electrodes. We used data recorded from 32 electrodes in the standard 32 channel placement. Activity from two (EOG) electrodes was also recorded but was ultimately not used in this work.

2 ARTIFACT CORRECTION

The artifact correction component consists of a stacked convectional encoder-decoder network. To create the training data we randomly extracted 104 samples from the clean EEG trials, the first and last $h = 32$ samples were stacked and used as the input to the model, the sample at position q from the remaining 40 samples was used as the ground truth. Essentially we are training a network to predict the qth sample from an occluded segment of 40 samples ($200ms$) using the $h = 32$ samples that before and after the removed segment. It is important to note that h was one of the hyper-parameters we optimized for. Additional hyper-parameters include the activation function, dropout parameters, the number and location of batch normalization layers, and the number and sizes of the convolutional layers, pooling layers, and filters. We determined the optimal topological configuration via a random search of the hyper-parameter space. In total we trained over 500 distinct architectural configurations for each q and retained only the best best performing network. This process is equivalent to performing similar optimization for an ensemble network that predicts all 40 segments, however our work-flow allows for easier parallelization of the training process. While it is impossible to describe each and every network in detail, the interested reader may find the best performing network for $q = 20$ in Figure S2.

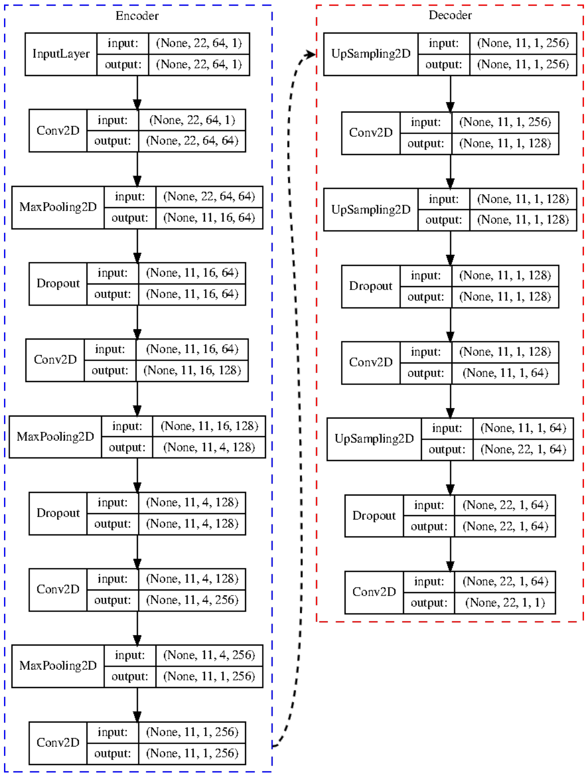

Figure S2. Our network, the dashed black arrow denotes an $11 \times 1 \times 256$ embedded data tensor. This embedded representation is passed form the encoder to the decoder which then reconstructs sample from the occluded segment.
